# The micropeptide LEMP plays an evolutionarily conserved role in myogenesis

**DOI:** 10.1038/s41419-020-2570-5

**Published:** 2020-05-11

**Authors:** Lantian Wang, Jing Fan, Lili Han, Haonan Qi, Yimin Wang, Hongye Wang, Suli Chen, Lei Du, Sheng Li, Yunbin Zhang, Wei Tang, Gaoxiang Ge, Weijun Pan, Ping Hu, Hong Cheng

**Affiliations:** 1 State Key Laboratory of Molecular Biology, Shanghai Key Laboratory of Molecular Andrology, Shanghai Institute of Biochemistry and Cell Biology, Center for Excellence in Molecular Cell Science, Chinese Academy of Sciences, University of Chinese Academy of Sciences, 200031 Shanghai, China; 2 State Key Laboratory of Cell Biology, Shanghai Institute of Biochemistry and Cell Biology, Center for Excellence in Molecular Cell Science, Chinese Academy of Sciences, University of Chinese Academy of Sciences, 200031 Shanghai, China; 3 Key Laboratory of Tissue Microenvironment and Tumor, CAS Center for Excellence in Molecular Cell Science, Shanghai Institute of Nutrition and Health, Shanghai Institutes for Biological Sciences, University of Chinese Academy of Sciences, Chinese Academy of Sciences, 200031 Shanghai, China; 4 Zebrafish Core Facility, Shanghai Institute of Biochemistry and Cell Biology, Center for Excellence in Molecular Cell Science, Chinese Academy of Sciences, University of Chinese Academy of Sciences, 200031 Shanghai, China; 5 Animal Core Facility, Shanghai Institute of Biochemistry and Cell Biology, Center for Excellence in Molecular Cell Science, Chinese Academy of Sciences, University of Chinese Academy of Sciences, 200031 Shanghai, China; 6 Institute for Stem Cell and Regeneration, Chinese Academy of Sciences, 100101 Beijing, China

**Keywords:** RNA, Muscle stem cells, Muscle stem cells

## Abstract

In recent years, micropeptides have been increasingly identified as important regulators in various biological processes. However, whether micropeptides are functionally conserved remains largely unknown. Here, we uncovered a micropeptide with evolutionarily conserved roles in myogenesis. RNA-seq data analysis of proliferating mouse satellite cells (SCs) and differentiated myotubes identified a previously annotated lncRNA, MyolncR4 (1500011K16RIK), which is upregulated during muscle differentiation. Significantly, MyolncR4 is highly conserved across vertebrate species. Multiple lines of evidence demonstrate that MyolncR4 encodes a 56-aa micropeptide, which was named as LEMP (lncRNA encoded micropeptide). LEMP promotes muscle formation and regeneration in mouse. In zebrafish, MyolncR4 is enriched in developing somites and elimination of LEMP results in impaired muscle development, which could be efficiently rescued by expression of the mouse LEMP. Interestingly, LEMP is localized at both the plasma membrane and mitochondria, and associated with multiple mitochondrial proteins, suggestive of its involvement in mitochondrial functions. Together, our work uncovers a micropeptide that plays an evolutionarily conserved role in skeletal muscle differentiation, pinpointing the functional importance of this growing family of small peptides.

## Introduction

Noncoding RNAs (ncRNAs) are important regulators in various biological and pathological processes^1–4^. Among these ncRNAs, most linear long ncRNAs (lncRNAs) have similar features to mRNAs, namely, they have both 5′ cap and 3′ polyA tail, and undergo splicing^5^. Despite of these structural similarities, lncRNAs are thought not to encode proteins, but instead function as microRNA sponges, scaffolds for recruiting proteins, and so on^6–10^. However, most recent work revealed that several originally annotated lncRNAs actually contain small open-reading frames (ORFs) and encode small peptides (<100-aa), also named as micropeptides, that play key roles in diverse biological processes^11–22^.

In general, lncRNAs are not conserved in nucleotide sequence. However, those encoding micropeptides usually show good conservation. Based on ribosome footprinting data and evolutionary conservation analysis, more than 60 translated small ORFs have been identified that contain conserved sequence in zebrafish and humans^23^. Despite of their sequence conservation, up to date, only a few micropeptides have been directly shown to play conserved roles^11,14,20^. It remains largely unclear whether the roles of micropeptides are generally conserved. Thus, the characterization of the biological functions of micropeptides displaying sequence conservation across species would be critical to understand whether micropeptides represent an important part of our proteome.

In this study, we identified a putative trans-membrane micropeptide, LEMP, which functions in myogenesis in both zebrafish and mice. LEMP expression increases along with myogenic differentiation. Consistent with this expression pattern, knockdown (KD) or knockout (KO) of LEMP in mouse myoblast impaired their differentiation, and LEMP KO mice show defective skeletal muscle formation and functions. In zebrafish, LEMP is enriched in somites during embryogenesis and plays an important role in muscle development. Notably, this role can be completely replaced by its mouse orthologue. Together, our study identifies LEMP as a micropeptide with evolutionarily conserved roles in myogenesis.

## Results

### Identification of MyolncR4 that is upregulated during myogenic differentiation

**Fig. 1:**
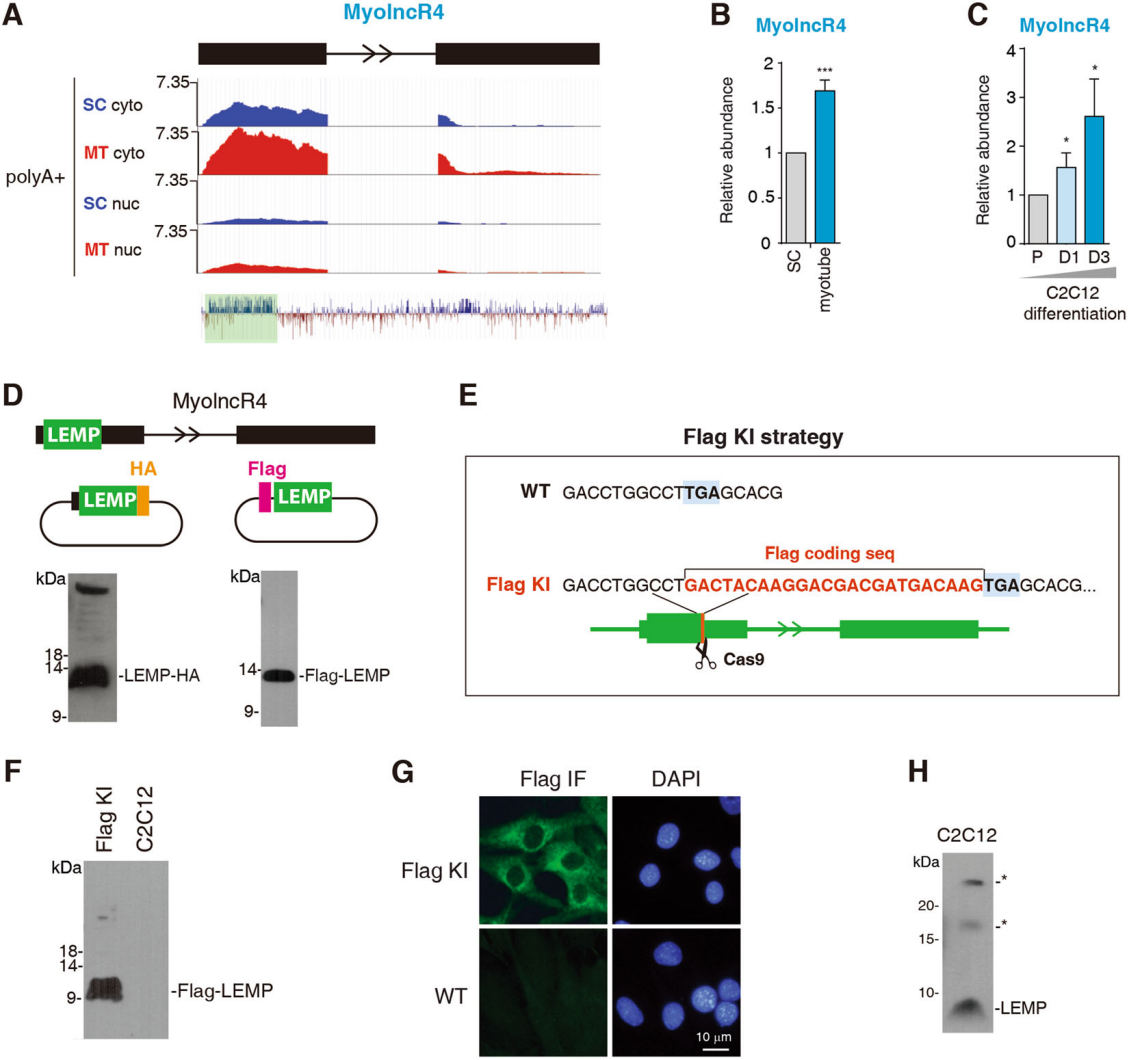
The expression of MyolncR4 increases along with myogenic differentiation. **a** Deep-sequencing signals of MyolncR4. Numbers to the left show the RPM. SC satellite cell, MT myotube, cyto cytoplasm, nuc nucleus. The regions are boxed in color is the most conserved part of MyolncR4. **b** RT-qPCRs to examine the expression level of MyolncR4 in satellite cells and differentiated myotubes. The bars show RNA levels relative to GAPDH. Error bars, standard deviations (*n* = 3). Statistical analysis was performed using Student’s *t* test. ***P < 0.001. **c** RT-qPCRs to examine the expression level of MyolncR4 during C2C12 differentiation. The bars show RNA levels relative to GAPDH. Error bars, standard deviations (*n* = 3). Statistical analysis was performed using Student’s *t* test. *P < 0.05. **d** (Top): Diagram of constructs used for transfection. (Bottom): Western blot to examine LEMP expression in HeLa cells transfected with LEMP-HA or Flag-LEMP. **e** Illustration of Flag knock-in strategy. **f**, **g** Western blot and IF to detect the expression of Flag-LEMP in Flag KI cells using the Flag antibody. **h** Western blot to examine the expression of endogenous LEMP with an antibody raised against the C-terminal region of LEMP. The asterisks indicate nonspecific bands that are detected by the LEMP antibody.

In order to identify lncRNAs involved in myogenesis, we carried out RNA-seq of proliferating satellite cells (SCs) and differentiated myotubes. As expected, many lncRNAs known to be enriched in myotubes were upregulated in our sequencing data (Supplementary Fig. S1). Among the newly identified lncRNAs upregulated in myotubes (MyolncRs, myogenic lncRNAs), MyolncR4, previously annotated as 1500011K16RIK, attracted our attention, as it is conserved from zebrafish to humans (Fig. 1a). The increased expression level of MyolncR4 in myotubes was confirmed by RT-qPCRs (Fig. 1b). To further validate the expression change of MyolncR4 during myogenic differentiation, we used C2C12 skeletal muscle myoblasts. Consistent with the observation obtained with primary cells, RT-qPCR data revealed the level of MyolncR4 was gradually elevated during the process of C2C12 differentiation (Fig. 1c).

### MyolncR4 encodes a 56-aa micropeptide

When looking into the most conserved part of MyolncR4, we immediately noticed a small ORF, putatively encoding a 56-aa micropeptide, which we named as LEMP (lncRNA-encoded micropeptide). Consistent with its translation possibility, MyolncR4 is mainly detected in the cytoplasm in our RNA-seq data (Fig. 1a). We used several strategies to examine its coding capacity. First, we cloned this lncRNA gene, including the putative 5′ UTR and the ORF, with an HA tag inserted upstream of the stop codon, into a eukaryotic expression vector (Fig. 1d, middle left). Alternatively, the putative ORF, with a Flag tag inserted upstream of the start codon, was cloned into the same expression vector (Fig. 1d, middle right). When these constructs were separately transfected into HeLa cells, western blot with an HA or Flag antibody detected the corresponding peptide, respectively (Fig. 1d, lower). To obtain more direct evidence for LEMP production, we used CRISPR-Cas9 to knock in (KI) a Flag tag at the MyolncR4 gene locus immediately before the stop codon in C2C12 cells (Fig. 1e). Successful KI was confirmed by DNA sequencing. Both western blot and immunofluorescence (IF) with the Flag antibody easily detected the expression of LEMP (Fig. 1f, g), further confirming the coding ability of the ORF in MyolncR4. We next generated a polyclonal antibody against LEMP. The antibody detected an endogenous peptide with the expected size in myoblast (Fig. 1h). Based on these data, we conclude that MyolncR4 encodes the micropeptide LEMP, while this work was in progress, three other groups independently identified this peptide^15,17,18^.

### The LEMP micropeptide promotes myogenic differentiation

**Fig. 2:**
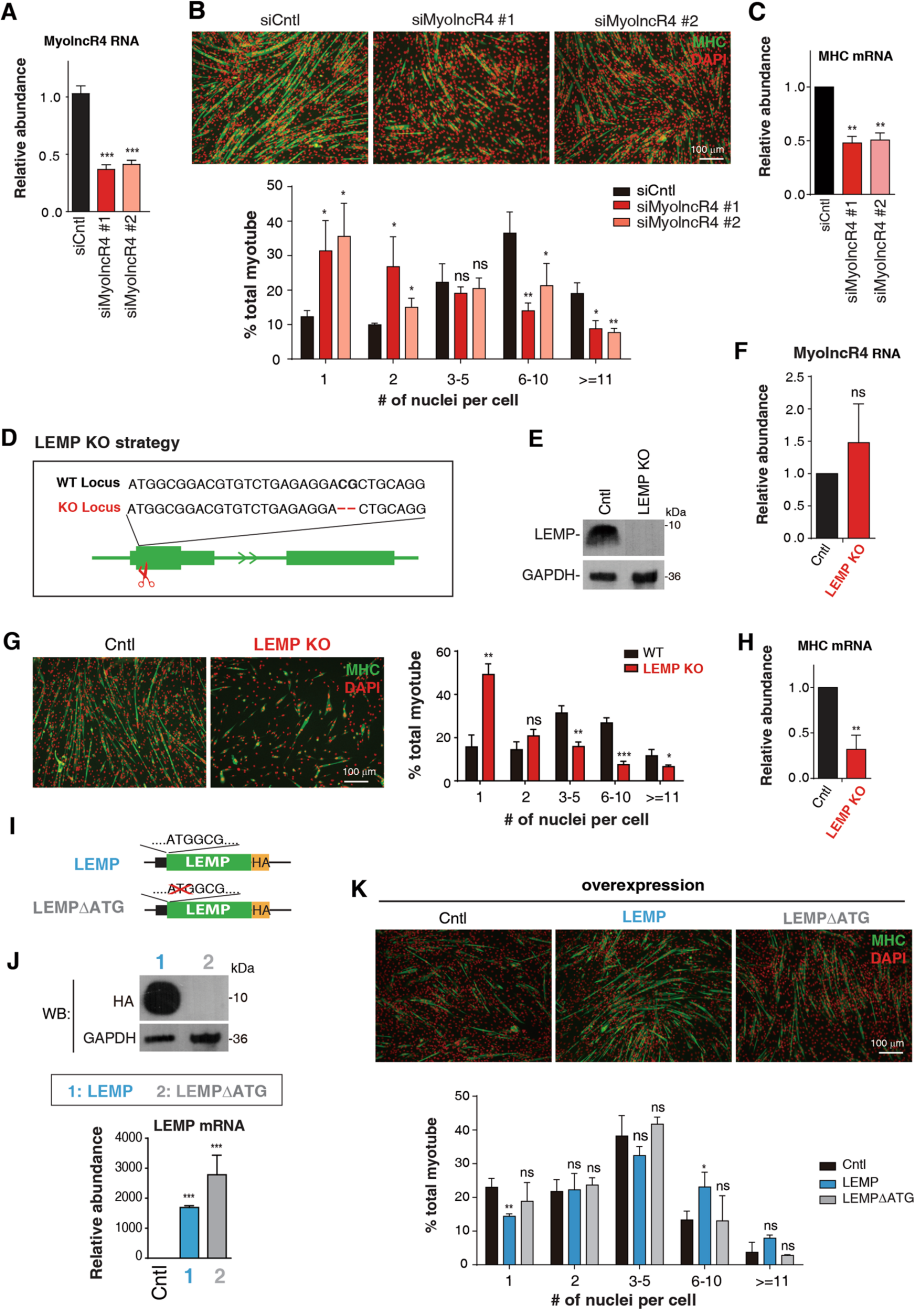
LEMP promotes myogenic differentiation. **a** RT-qPCRs to examine MyolncR4 knockdown efficiency. The bars show RNA levels relative to GAPDH. Error bars, standard deviations (*n* = 3). Statistical analysis was performed using Student’s *t* test. ***P < 0.001. **b** (Top): IF with the MHC antibody to examine differentiation of C2C12 transfected with Cntl and MyolncR4 siRNAs. DAPI staining to mark the nuclei. MHC: green; DAPI: red. Scale bar, 100 μm. (Bottom): Quantitation of the number of nuclei present in myotubes. Error bars, standard deviations (*n* = 3). Statistical analysis was performed using Student’s *t* test. **P < 0.01, *P < 0.05, ns: not significant. **c** RT-qPCRs to examine MHC mRNA levels in MyolncR4 knockdown cells. The bars show RNA levels relative to GAPDH. Error bars, standard deviations (*n* = 3). Statistical analysis was performed using Student’s *t* test. **P < 0.01. **d** Illustration of the LEMP KO strategy. **e** Western blot to examine LEMP expression in Cntl or LEMP KO cells. GAPDH was used as a loading control. **f** RT-qPCRs to examine MyolncR4 RNA levels in Cntl and LEMP KO cells. The bars show RNA levels relative to GAPDH. Error bars, standard deviations (*n* = 3). Statistical analysis was performed using Student’s *t* test. Ns: not significant. **g** (Left): IF with the MHC antibody to examine differentiation of Cntl or LEMP KO cells. DAPI staining to mark the nuclei. MHC: green; DAPI: red. Scale bar, 100 μm. (Right): Quantitation of the number of nuclei present in myotubes. Error bars, standard deviations (*n* = 3). Statistical analysis was performed using Student’s *t* test. ***P < 0.001, **P < 0.01, *P < 0.05, ns: not significant. **h** RT-qPCRs to examine MHC mRNA levels in Cntl and LEMP KO cells. The bars show RNA levels relative to GAPDH. Error bars, standard deviations (*n* = 3). Statistical analysis was performed using Student′s *t* test. **P < 0.01. **i** Schematic of the LEMPΔATG construct. The start codon of LEMP was deleted. **j** Western blot and RT-qPCRs to examine LEMP protein and mRNA levels in C2C12 cells transfected with equal amount of Cntl, LEMP-HA, and LEMPΔATG-HA constructs. **k** (Top): IF with the MHC antibody to examine differentiation of C2C12 cells transfected with plasmid expressing LEMP, LEMPΔATG at 4 days after differentiation induction. DAPI staining to mark the nuclei. MHC: green; DAPI: red. Scale bar, 100 μm. (Bottom): Quantitation of the number of nuclei per myotubes. Error bars, standard deviations (*n* = 3). Statistical analysis was performed using Student’s *t* test. ***P < 0.001, **P < 0.01, *P < 0.05, ns: not significant.

To investigate the function of MyolncR4, we first treated C2C12 cells with two different pieces of siRNAs to knock down its expression. These siRNAs efficiently reduced the expression level of MyolncR4 by 60–70% (Fig. 2a). As expected, C2C12 myoblasts treated with Cntl siRNA differentiated normally (Fig. 2b). In contrast, MyolncR4 KD cells showed myotube formation defects after induction of differentiation (Fig. 2b). Consistent with the morphological changes, the expression level of myosin heavy chain (MHC) was reduced in MyolncR4 KD cells (Fig. 2c), suggesting an indispensable role of LEMP in muscle differentiation.

To examine whether the differentiation defects were caused by the loss of LEMP peptide, or by the reduction in MyolncR4 RNA level, we generated LEMP KO myoblast cell line by disrupting the reading-frame of LEMP using CRISPR-Cas9 (Fig. 2d). This KO strategy eliminated expression of the LEMP micropeptide without affecting the RNA level of MyolncR4 (Fig. 2e, f). Although control cells differentiated normally, LEMP KO cells showed obvious differentiation defects and significantly reduced MHC levels (Fig. 2g, h). These data indicate that the LEMP peptide, rather than the MyolncR4 RNA, is required for myoblast differentiation.

We next examined whether overexpression of LEMP promotes myoblast differentiation. C2C12 cells were transfected with a plasmid overexpressing LEMP. A mutant plasmid (LEMPΔATG), in which the LEMP start codon was deleted, was also constructed and overexpressed (Fig. 2i). Note that ATG deletion in LEMPΔATG completely blocked LEMP production, but did not reduce the RNA level (Fig. 2j). Four days after differentiation induction, cells overexpressing LEMP displayed better differentiation abilities, as compared to control cells transfected with an empty plasmid (Fig. 2k). In contrast, LEMPΔATG overexpression did not show similar differentiation promoting effects (Fig. 2k). These results suggest that the LEMP micropeptide, instead of the MyolncR4 RNA, plays the major role to promote myoblast differentiation.

### LEMP KO mice show muscle formation defect and muscle weakness

**Fig. 3:**
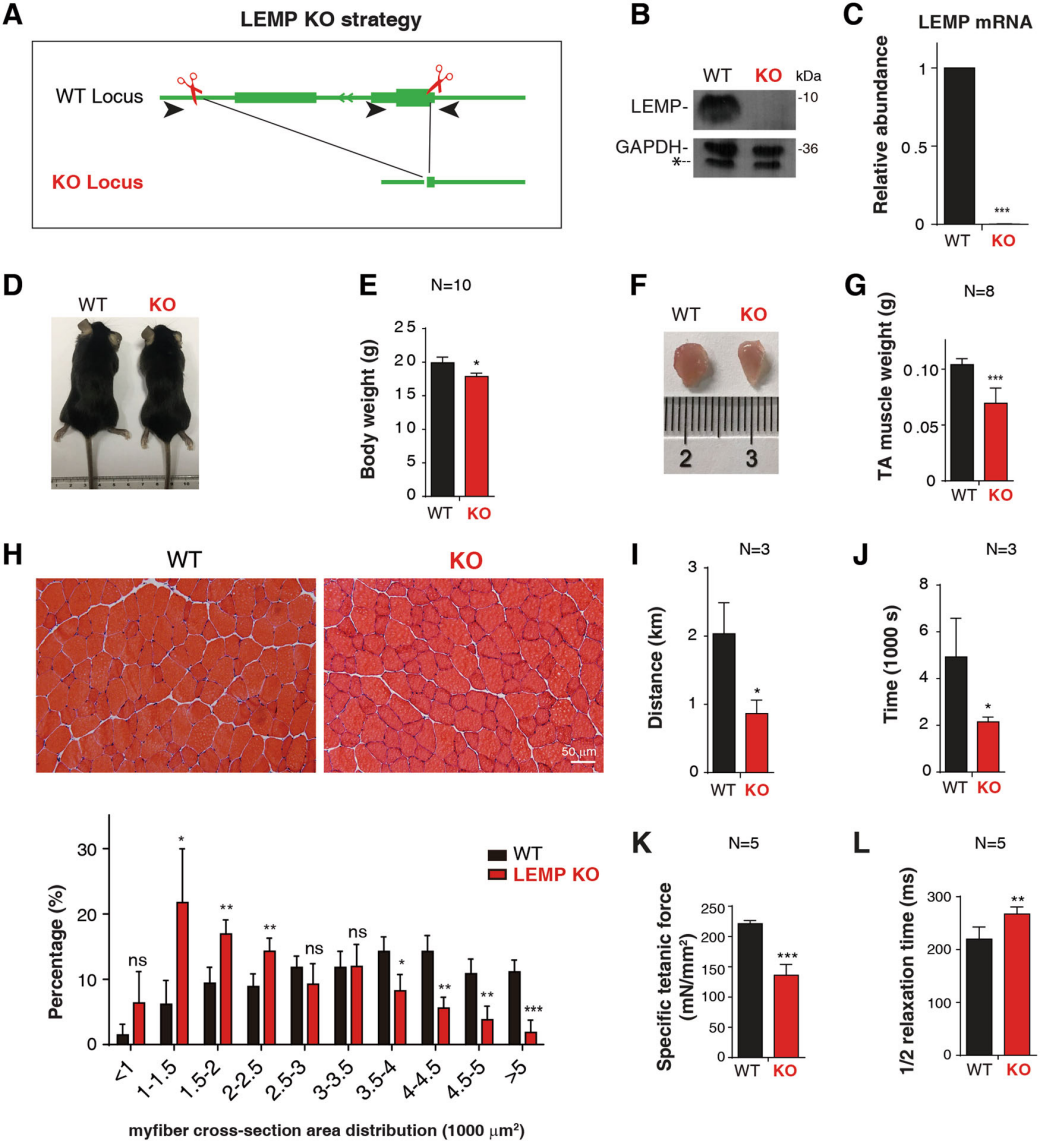
LEMP is essential for muscle development in mice. **a** Strategy for LEMP KO mice using CRISPR-Cas9 genome editing. **b** Western blot to examine expression levels of LEMP protein in satellite cells isolated from WT and LEMP KO mice. GAPDH was used as a loading control. The asterisk indicates a nonspecific band that is detected by the GAPDH antibody. **c** RT-qPCRs to examine expression levels of LEMP mRNA in satellite cells isolated from WT and LEMP KO mice. The bars show RNA levels relative to GAPDH. Error bars, standard deviations (*n* = 3). Statistical analysis was performed using Student’s *t* test. ***P < 0.001. **d** A representative image of 8-week-old WT and LEMP KO mice. **e** Quantification of the body weight from 8-week-old WT and LEMP KO mice. Error bars, standard deviations (*n* = 10). Statistical analysis was performed using Student’s *t* test. *P < 0.05. **f** A representative image of TA isolated from 8-week-old WT and LEMP KO mice. **g** Quantification of TA weight from 8-week-old WT and LEMP KO mice. Error bars, standard deviations (*n* = 8). Statistical analysis was performed using Student’s *t* test. ***P < 0.001. **h** (Top): Representative images of H&E staining to examine myofiber sizes of TA muscle cross-sections from 8-week-old WT and LEMP KO mice. Scale bar, 50 μm. (Below): Percentage distribution of muscle fiber cross-section area derived from muscles from WT and LEMP KO mice. Error bars, standard deviations (*n* = 4). Statistical analysis was performed using Student’s *t* test. ***P < 0.001, **P < 0.01, *P < 0.05, ns: not significant. **i**, **j** Measurement of exercise capacity using forced treadmill running to exhaustion. Distance **i** and time **j** are shown. Error bars, standard deviations (*n* = 3). Statistical analysis was performed using Student’s *t* test. *P < 0.05. **k** Specific tetanic force of TA muscles in WT or LEMP KO mice. Error bars, standard deviations (*n* = 5). Statistical analysis was performed using Student’s *t* test. ***P < 0.001. **l** 1/2 relaxation time of TA muscles in WT or LEMP KO mice. Error bars, standard deviations (*n* = 5). Statistical analysis was performed using Student’s *t* test. **P < 0.01.

To investigate the function of LEMP in vivo, we generated LEMP KO mice using CRISPR-Cas9 to remove its entire coding region and 3′ UTR (Fig. 3a). The LEMP KO mice were born normally at the Mendelian frequency, demonstrating that LEMP is not essential for embryonic development. Efficient LEMP KO was confirmed by western blot and RT-qPCRs (Fig. 3b, c). The sizes and weights of LEMP KO mice were moderately reduced compared to those of the wild type (WT) littermates (Fig. 3d, e). Notably, the sizes and weights of the tibialis anterior (TA) muscles were also reduced (Fig. 3f, g). Consistent with the lower body weight, HE staining showed that the cross-section area of TA muscle fibers significantly decreased in KO mice compared to that of WT (Fig. 3h), suggesting potential muscle development defect. The LEMP KO mice also showed weakened muscle functions, evident by reduced abilities in exhaustive running (Fig. 3i, j). In further support of impaired muscle functions, the specific titanic force was decreased in LEMP KO mice (Fig. 3k), and the 1/2 relaxation time of the LEMP KO mice TA muscles was increased (Fig. 3l). Thus, LEMP is important for myogenesis in mouse.

### LEMP promotes skeletal muscle regeneration

**Fig. 4:**
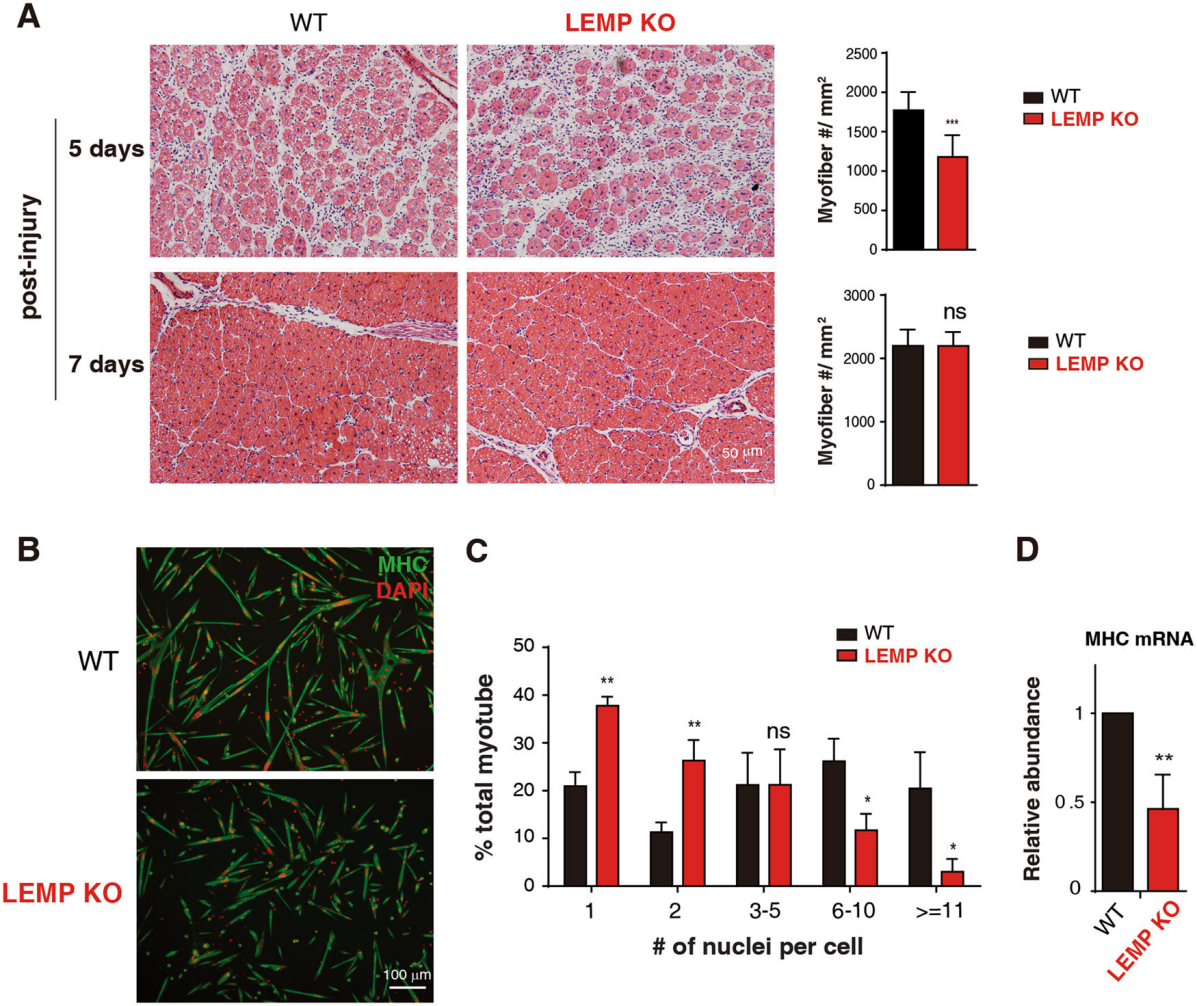
LEMP promotes skeletal muscle regeneration and activates satellite cell differentiation. **a** (Left): Representative images of H&E-staining of TA muscle cross-sections at day 5 and day 7 post injury from WT and LEMP KO mice. Scale bar, 50 μm. (Right): Statistics on the number of regeneration fibers per unit area. Error bars, standard deviations (*n* = 10). Statistical analysis was performed using Student’s *t* test. ***P < 0.001. **b** MHC immunostaining of satellite cells isolated from WT and LEMP KO mice. MHC: green; DAPI: red. **c** Quantitation of fusion in WT and LEMP KO satellite cells. Scale bar, 100 μm. Error bars, standard deviations (*n* = 3). Statistical analysis was performed using Student‘s *t* test. **P < 0.01, *P < 0.05, ns: not significant. **d** RT-qPCRs to examine MHC mRNA expression level in satellite cells isolated from WT and LEMP KO mice. The bars show RNA levels relative to GAPDH. Error bars, standard deviations (*n* = 3). Statistical analysis was performed using Student’s *t* test. **P < 0.01.

Regeneration of injured adult skeletal muscle involves differentiation of activated SCs to form new myofibers^24–27^. To investigate the role of LEMP in muscle regeneration, we injected cardiotoxin (CTX) into the TA muscle to induce muscle injury in WT and LEMP KO mice. Notably, compared to that in WT mice, HE staining of TA muscle cross-sections in the LEMP KO sample showed reduced numbers of myofibers at 5-day post-injury and no statistical difference in myofibers number at 7-day post-injury (Fig. 4a). This result indicates that LEMP is required for rapid and efficient muscle regeneration in adult.

To examine whether the regeneration defect observed in LEMP KO mice is due to impaired SC differentiation, we next isolated SCs from WT and LEMP KO mice. Indeed, SCs isolated from LEMP KO mice displayed reduced differentiation abilities compared to those from WT mice (Fig. 4b, c). Consistent with cell morphological results, the expression of MHC was also decreased in myotubes differentiated from LEMP KO SCs (Fig. 4d). These data together suggest that LEMP promotes muscle regeneration by promoting differentiation of SCs.

### LEMP is important for muscle development in zebrafish

**Fig. 5:**
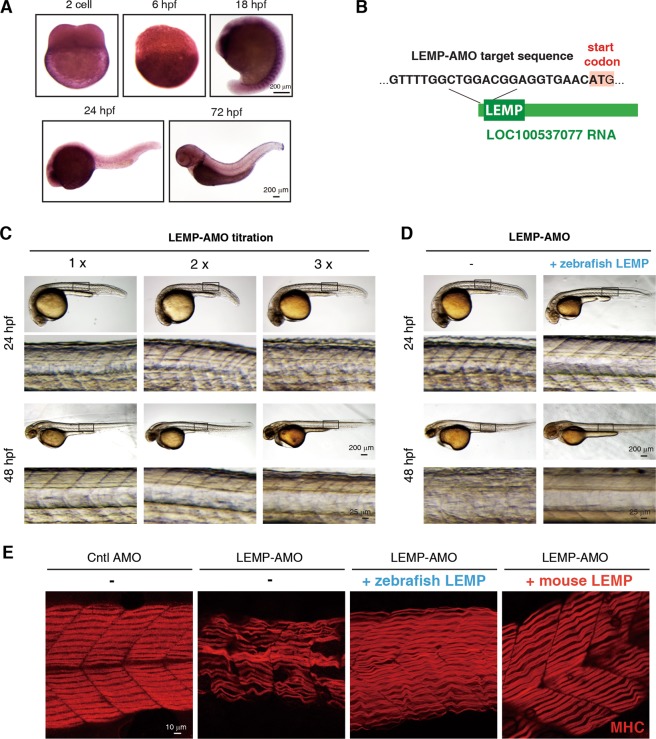
LEMP is important for muscle development in zebrafish. **a** In situ hybridization to examine the localization of MyolncR4 at different developmental stages of zebrafish embryos with a specific probe targeting zLEMP. hpf, hours post fertilization. Scale bar, 200 μm. **b** Diagram to show the targeting sequence of the LEMP-AMO. **c** Representative images of zebrafish at 24 or 48-hpf after injection of different amounts of LEMP-AMO at one-cell stage. Scale bar, 200 μm or 25 μm. **d** Representative images of zebrafish at 24 or 48-hpf after injection of LEMP-AMO in the presence or absence of the zebrafish LEMP mRNA at one-cell stage. Scale bar, 200 or 25 μm. **e** MHC immunostaining to examine myofiber alignment in 48-hpf embryos injected with Cntl-AMO, LEMP-AMO, LEMP-AMO with zLEMP mRNA, or LEMP-AMO with mLEMP mRNA, respectively. Scale bar, 10 μm.

We next sought to investigate whether the functions of LEMP in muscle development are evolutionarily conserved. Considering that LEMP is highly conserved from zebrafish to humans, we studied its functions in muscle in zebrafish. To investigate the expression pattern of this putative LEMP orthologue in zebrafish, we carried out in situ hybridizations with a specific probe targeting zebrafish LEMP (zLEMP) in embryos at two-cell stage, as well as those at 6, 18, 24, and 72 h post-fertilization (hpf) (Fig. 5a). Notably, at 18 hpf, intense expression of LEMP was exclusively observed in the developing somites (Fig. 5a), suggesting its involvement in muscle development.

We next used an antisense morpholino oligo (LEMP-AMO), which hybridizes to the start codon region of zLEMP to disrupt its expression (Fig. 5b). To examine the effect of LEMP-AMO, we constructed an EGFP reporter plasmid (LAT-EGFP, LEMP-AMO targeting EGFP), in which the LEMP-AMO targeting sequence was inserted at the corresponding region of EGFP (Supplementary Fig. S2). When this plasmid was injected alone, or co-injected with a Cntl AMO, which does not target any specific sequence in zebrafish, into the embryo, GFP fluorescence signal was easily detected (Supplementary Fig. S2). In contrast, when co-injected with the LEMP-AMO, EGFP expression of LAT-EGFP was completely abolished (Supplementary Fig. S2), indicating that the LEMP-AMO is effective. We then examined how reduction of LEMP expression affects zebrafish muscle development by injecting different amounts of LEMP-AMO into the fish embryos at 1-cell stage. Along with the increased AMO amounts, the morphants showed increasingly misaligned muscle fibers at 24 and 48 hpf (Fig. 5c), indicative of muscular development defects.

To determine whether these defects are due to the loss of LEMP expression, we performed the rescue assay by re-expression of the zLEMP mRNA in the same embryo carrying LEMP-AMO. We found that the LEMP mRNA fully rescued the LEMP inhibition morphant phenotypes (Fig. 5d). Taken together, we conclude that LEMP is required for zebrafish skeletal muscle development and the role of LEMP in myogenesis is conserved.

### The mouse LEMP (mLEMP) efficiently substitutes the function of its zebrafish orthologue

To further investigate the functional conservation of LEMP, we next examined whether the mLEMP could substitute zLEMP for its function in muscle development. To do this, we co-injected the mRNA encoding mLEMP together with LEMP-AMO into zebrafish embryos at 1-cell stage, using the zLEMP mRNA as a control. At 48 hpf, MHC IF staining was performed to examine the muscle fiber alignment. Consistent with the observation obtained with the optical microscope (Fig. 5c, d), confocal microscopic data demonstrate that injection of the LEMP-AMO itself resulted in significantly misaligned muscle fibers, and co-injection of the zLEMP mRNA rescued these defects (Fig. 5e). Importantly, co-injection of the mLEMP mRNA also fully rescued the muscle fiber misalignment caused by inhibition of zLEMP (Fig. 5e). These data further demonstrate the functional conservation of LEMP and indicate that LEMP promotes muscle formation in zebrafish and mouse through similar mechanisms.

### LEMP localizes at plasma membrane and mitochondria and associates with multiple mitochondrial proteins

**Fig. 6:**
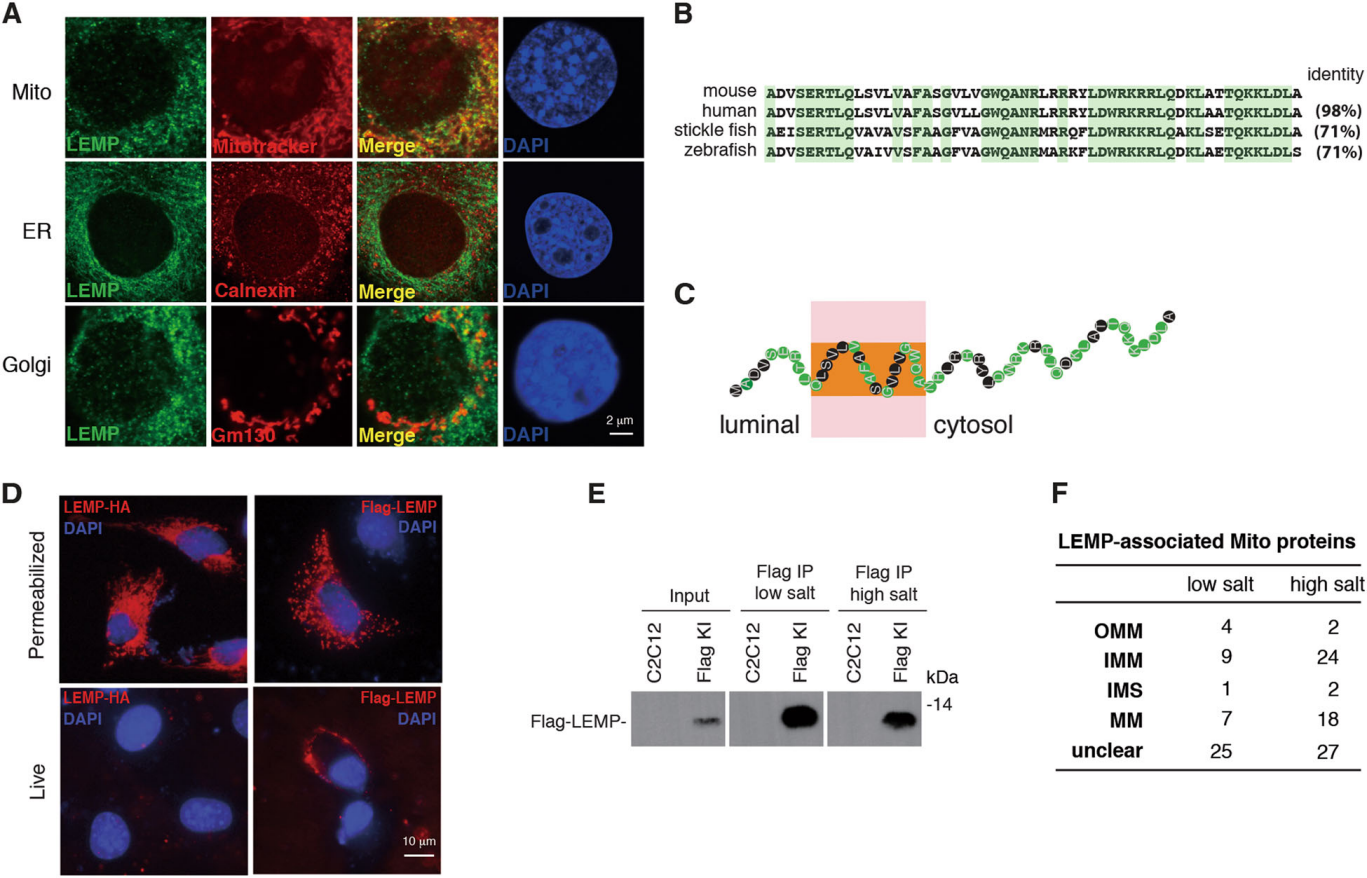
LEMP localizes at the plasma membrane and mitochondria and associates with mitochondrial proteins. **a** IF using a Flag antibody to examine the localization of LEMP in Flag-LEMP-KI C2C12 cells. Mitotracker, Calnexin, and Gm130 were stained to show the localization of mitochondrion, ER, and Golgi. Scale bar, 2 μm. **b** Amino acid sequence alignment of LEMP proteins in multi-species. Conserved amino acids are marked in green. **c** Model of LEMP topology based on TMHMM Server v. 2.0. **d** IF using an HA or a Flag antibody to examine the localization of LEMP-HA and Flag-LEMP in permeabilized (fixed) or nonpermeabilized (live) C2C12 cells. Scale bar, 10 μm. **e** IPs with the Flag antibody to identify LEMP-associating proteins from Flag-LEMP cells in the presence of RNaseA under low salt (150 mM) and high salt (300 mM) conditions followed by western blot and mass spectrum. **f** The category of LEMP-associated mitochondrial proteins. OMM outer mitochondrial membrane, IMM inner mitochondrial membrane, IMS intermembrane space, MM mitochondrial matrix.

To obtain some insights into the mechanism for LEMP promoting myogenic differentiation, we used Flag-LEMP-KI cells to study its subcellular localization. Confocal microscopy analysis revealed that LEMP apparently co-localized with the mitochondria marker mitotracker and partly co-localized with the Golgi maker Gm130, but not the endoplasmic reticulum (ER) marker Calnexin, demonstrating that it is mainly localized in mitochondria (Fig. 6a). The amino acid sequence of LEMP is highly conserved from zebrafish to human, especially for the C terminus (Fig. 6b). Based on TMHMM Server v. 2.0, the relatively less conserved N terminus was predicted to contain a transmembrane domain with the N terminus facing to the luminous (Fig. 6c). Indeed, when Flag-LEMP and LEMP-HA were separately expressed in C2C12 cells, live cell IF staining showed that the N terminal Flag, but not the C terminal HA, was detected at the plasma membrane in non-permeabilized cells, although they show similar staining signals and patterns in fixed cells (Fig. 6d).

We next took advantage of the Flag-LEMP-KI cells to carry out Flag immunoprecipitation for the identification of LEMP-associating proteins under low (150 mM) or high (300 mM) salt conditions (Fig. 6e). Consistent with its mitochondrial localization, many mitochondrial proteins were identified under both conditions (Fig. 6f, DataSet 1). Interestingly, compared to those in low salt condition, more inner membrane (IMM) and membrane matrix (MM) proteins were associated with LEMP in high salt condition (Fig. 6f, DataSet 1), suggesting that the associations of LEMP with IMM and MM proteins might be tighter than outer mitochondrial membrane (OMM) proteins.

## Discussion

In this study, we identified a 56-aa micropeptide LEMP encoded by a lncRNA, which promotes myogenesis both in embryonic stage and in adult and highlights its functional conservation. Interestingly, LEMP localizes to mitochondria and tightly associates with multiple mitochondrial proteins, suggesting that it might regulate muscle development and regeneration by modulating mitochondria functions. In addition, we also observed that LEMP could partly co-localize with the Golgi. LEMP might have some unknown function in Golgi which needs further careful studies.

In recent years, several micropeptides encoded by previously annotated lncRNAs have been identified^11–22^. Here, we provide multiple lines of evidence that LEMP is another new case both in vitro and in vivo. Importantly, although we cannot completely exclude the possibility that MyolncR4 has some noncoding functions, all the data here point to the view that it promotes myogenic differentiation mainly by encoding the LEMP peptide. Together with other studies on lncRNA-encoded peptide, our work suggests that previously annotated lncRNAs should be carefully reconsidered for their coding potentials, especially for those evolutionarily conserved and distributed in the cytoplasm. Re-analysis of lncRNAs with the above features might lead to discoveries of more micropeptides with important biological functions.

Although many micropeptides have been identified up to date, only a few of them have been shown to be functionally conserved^11,14,20^. Our study provides direct evidence that LEMP is a micropeptide with evolutionarily conserved function in myogenesis. LEMP is highly conserved from mammals to zebrafish, sharing 71% amino acid identity between these species. Loss-of-function of LEMP in both mice and zebrafish resulted in muscle development defects and function disruption. Importantly, the mLEMP completely substituted the function of its zebrafish orthologue, suggesting that they promote muscle development in these two species through similar mechanisms. Thus, our study supports the view that unlike lncRNAs, micropeptides in general are functionally conserved, pinpointing the functional importance of these growing family of small peptides.

LEMP mainly localizes in mitochondria and tightly associates with multiple mitochondrial proteins, especially IMM and MM proteins, raising the possibility that LEMP promotes muscle function by regulating mitochondria functions. The reduced exhaustive running ability of LEMP KO mice is consistent with dysregulation of mitochondria. In further support of this view, the most recent studies by other groups also provided evidence that KO of LEMP in mice disrupted mitochondria functions. It is interesting to further explore how LEMP KO led to mitochondria defects and reduced muscle functions. Also, whether LEMP promotes SC differentiation through regulating mitochondria function and the mechanism behind also requires future investigation. Except for mitochondria, LEMP also localizes at the plasma membrane, similar to the localization of myomaker which is required for myoblast fusion^28–30^. LEMP may also have functions in myoblast fusion during differentiation. It will be an interesting topic for future studies.

Interestingly, including LEMP, many micropeptides so far identified are involved in muscle functions^12,14,15,18,20–22^. Another intriguing observation with these micropeptides is their membrane localization. Further studies on the identification and functional characterization for more micropeptides are required to understand whether membrane localization is a common feature for micropeptides and the functional relevance of this feature.

## Materials and methods

### Plasmids and antibodies

To construct the LEMP-HA, Flag-LEMP plasmids, the corresponding sequence was HA or Flag tagged and inserted into the pHAGE-fEF1a-IRES-ZsGreen vector. LEMPΔATG-HA plasmid was obtained using mutagenesis. To construct LEMP-Flag-px330, a single-guide RNA (sgRNA) specific to the C-terminal coding sequence of mLEMP locus was cloned into the px330. The donor plasmid was made with a Flag tag in-frame with the LEMP coding sequence and flanked by ~500 base pair homology arms specific to the LEMP locus. The zebrafish cDNA of LEMP gene was amplified and cloned into pCS2+ vector. To construct the plasmid used for synthesis of antisense RNA probe, the relevant sequence of LEMP was cloned into pCS2+ vector. To make the construct of LAT-EGFP, the zLEMP-AMO targeting sequence was inserted into the corresponding start codon region of EGFP in pCS2+ vector.

The antibodies against Flag (Sigma, F3165), HA (Sigma, H6908), GAPDH (Abcam, ab8245), MHC (Upstate, 05-715), Mitotracker (Invitrogen, M7510), Gm130 (BD, 610823), Calnexin (Santa Cruz, SC-6465), LEMP (ABclonal, A18310), CD31 (BD, 562861), CD45 (eBioScience, 45-0451-82), CD11B (eBioScience, 45-0112-82), Sca1 (eBioScience, 56-5981-82), CD34 (BD, 553733), and integrin-α-APC (R&D, FAB3518A) were purchased.

### Conventional WISH in situ analysis

The LEMP probes were transcribed in vitro by T3 or T7 polymerase (Ambion) with Digoxigenin RNA Labelling Mix (Roche). Conventional whole-mount in situ hybridization (WISH) was described previously^31^. Images of conventional WISH were mounted in 4% methylcellulose and captured by Olympus SZX16 microscope with Olympus DP80 CCD.

### Cell culture, differentiation, and transfection

SCs from LEMP WT and KO mice were isolated as previously described^32^. Briefly, SCs were cultured on collagen-coated dishes in F10 basal medium (F10 medium containing 10% FBS and 2.5 ng/mL FGF), and cytokine cocktail medium (F10 medium containing 10% FBS, 5 ng/mL IL-1α, 5 ng/mL IL-13, 10 ng/mL IFN-Υ, 10 ng/mL TNF-α, and 2.5 ng/mL FGF). Then the cells were differentiated in differentiation medium (DMEM medium containing 2% horse serum) for 2 days. C2C12 myoblast cells were cultured in Dulbecco’s modified Eagle’s medium (DMEM) containing 10% FBS and penicillin/streptomycin, and differentiated in differentiation medium for 4 days.

DNA transfection was performed using Lipofectamine LTX (Invitrogen) following manufacturer’s instructions. The sequence of sgRNAs are shown in Table S1. For RNAi, siRNAs were transfected with Lipofectamine RNAiMax (Invitrogen) according to the manufacture’s protocols. In order to attain the best KD efficiency, the process was repeated after the first KD 24 h later. The siRNA-targeting sequences are shown in Table S1.

### Immunofluorescent analysis of cultured cells

Cells were fixed with 4% PFA for 15 min and permeabilized with 0.1% Triton in 1x PBS for 15 min. Fixed and permeabilized cells were incubated with primary antibody diluted in blocking buffer (1x PBS, 0.1% Triton X-100, and 2 mg/mL BSA) for 1 h at room temperature. Cells were washed three times with 1x PBS and incubated with Alexa Fluor 546-labeled or 488-labeled antibody for another 1 h, followed by DAPI staining.

For live cell staining, non-permeabilized cells were preblocked with blocking buffer (3% BSA in 1x PBS) for 15 min, and incubated with the primary antibody in the same buffer for 1 h on ice. After primary antibody incubating and washing, cells were fixed with 4% PFA and washed before the addition of secondary antibody. Cells were incubated with Alexa Fluor 546-labeled secondary antibody for 1 h, followed by DAPI staining. Fluorescence microscopic images were captured with a DP72 charge-coupled device camera on IX71 microscope using DP-BSW software (Olympus) or were acquired by Olympus FV1200 confocal microscope.

### SCs isolation

SCs were isolated as previously described^32^. Briefly, hind leg muscles from 2-month-old mice were dissected and dissociated with collagenase. The cell suspension was filtered through a 70 μm nylon filter (Falcon) and incubated with the CD45, CD11b, CD31, Sca1, CD34-FITC, and integrin-α-APC antibodies. The viable CD34^+^ integrin-α7^+^ SCs in the negative population of CD45, CD11b, CD31, and Sca1 cells were collected by FACS sorting. For serial expansion, 1 × 10^4^ SCs were seeded in 3.5 cm collagen-coated dishes, and the cell passage is carried out every 2 days.

### Treadmill exercise

Mice were run on Exer-3/6 treadmill apparatus with mild electrical stimulus. The treadmill was set to ramp from 0 to 10 m/min over a period of 5 min and then stay at 10 m/min for an additional 10 min. The treadmill speed then increased (2 m/min every 2 min) to a maximum speed of 30 m/min until exhaustion. Exhaustion was defined by the failure to run for longer than 10 s.

## Supplementary information

supplemental information

Dataset 1
