# Supplementary material for: The micropeptide LEMP plays an evolutionarily conserved role in myogenesis
Source: Cell Death Dis. 2020 May 11;11(5):357. doi: 10.1038/s41419-020-2570-5 (PMC7214441; doi:10.1038/s41419-020-2570-5)
Supplement: Supplementary file 1 — supplemental information [file 41419_2020_2570_MOESM1_ESM.docx]

**The micropeptide LEMP plays an evolutionarily conserved role in myogenesis**

Lantian Wang^1,#^, Jing Fan^1,#^, Lili Han^2, #^, Haonan Qi^3,#^, Yimin Wang^1^, Hongye Wang^2^, Suli Chen^1^, Lei Du^1^, Sheng Li^2^, Yunbin Zhang^5^, Gaoxiang Ge^2^, Weijun Pan^3,*^, Ping Hu^2,4*^, Hong Cheng^1,*^

**Inventory of supplementary data**

| Page | Description |
| --- | --- |
| 1 | Cover Page |
| 1-5 | Supplementary Methods |
| 6 | Figure S1 |
| 7 | Figure S2 |
| 7-8 | Supplemental Figure Table |

**Supplementary Methods and Materials**

**Animals**

All mouse work described in this manuscript has been approved and conducted under the Institutional Animal Care and Use Committee at Shanghai Institute of Biochemistry and Cell Biology. All mouse lines were generated on a pure C57BL/6N background. All functional experiments were performed using 6- to 8-week-old mice. The LEMP KO mouse was generated using CRISPR-Cas9 system to deplete the entire coding region and 3’ UTR of LEMP.

The zebrafish facility and study were approved by the Animal Research Advisory Committee of Institute of Nutrition and Health, SIBS, CAS, and zebrafish were maintained according to the guidelines of the Institutional Animal Care and Use Committee.  The Tubingen wild-type strains were used in this study.

**Whole-Mount Immunohistofluorescent Staining**

Embryos were fixed in 4% paraformaldehyde overnight at 4 ^0^C, followed by dehydrated and rehydrated with methanol/PBST series. Embryos were incubated with cold acetone for 20 min and washed 3 times with 1 x PBST, followed by proteinase K digestion. The embryos were blocked in blocking buffer and incubated with primary antibody against MHC diluted in incubating buffer (1 x PBS, 0.3% Triton X-100, 10 mg/mL BSA, 1% DMSO, 2% goat serum) for overnight. Embryos were washed 3 times with incubating buffer and incubated with Alexa Fluor 546-labeled secondary antibody for overnight, followed by DAPI staining. All images were acquired on Olympus FV1200 confocal microscope.

**MO/mRNA microinjection**

Morpholino oligonucleotides (MOs) were ordered from Gene Tools, LLC. Capped mRNAs were transcribed from linearized pCS2+ plasmids (mMessage Machine; Ambion), purified and diluted to 200 ng/μL for microinjection into zebrafish embryos at 1-cell stage.

**RNA Extraction and RT-qPCRs**

Total RNA was extracted using TRIzol (Invitrogen) and treated with RNase-free RQ1 DNase I (Promega) for 2 hr at 37 ^0^C to remove genomic DNA. Random primer was used for reverse transcription by M-MLV reverse transcriptase (Promega). Quantitative PCR was carried out using Go Taq Master Mix (Promega) according to the manufacture’s protocol. The primers used for PCR are listed in **Table S2**.

**Myofiber cross section area measurement**

The myofiber cross section area was measured with Image J software. Four independent visual fields were chosen randomly in each sample and 250 myofibers were measured from each field for analysis.

**Protein Immunoprecipitations**

For each assay, cells were harvested and re-suspended in the lysis buffer (20 mM Tris-HCl pH7.4, 150 mM NaCl or 300 mM NaCl, 2 mM EDTA pH 8.0, 0.1% Triton, 1 mM DTT, 1 mM PMSF). After sonication and centrifugation, the lysates were treated with RNase A for 20 min at 30 ^0^C, followed by incubation with Flag antibody-crosslinked beads at 4 ^0^C overnight. The beads were washed with the lysis buffer for four times and eluted with elution buffer (0.2M Glycine, 0.15% NP-40 pH 2.3). The samples were eluted and used for Mass Spectrometry.

**In vivo muscle force analysis**

*In vivo* TA muscle force analysis was performed with 1300 A 3-in-1 whole animal system (Aurora Scientific). Mice were anesthetized by 3-bromo-2-fluoro-phenyl methanol. The hind limb was shaved and immobilized by fixing the leg in a frame without disturbing the blood flow. A differential electrode arrangement with one EMG electrode was placed in the belly of the muscle, one near where the muscle inserts into the tendon, and one adjacent to the site of stimulation.  The distal TA tendon suture loop was attached to the lever arm hook of the instrument and measured the contractile force.  The results were analyzed by DMA software (Aurora scientific). For each treatment, ten independent experiments were performed.

**RNA-seq**

For polyA+ RNA sequencing, 5 μg of nuclear and cytoplasmic RNA was prepared from proliferating satellite cells or differentiated myotube cells with TRIzol, and used for polyA+ RNA selection. Stranded cDNA libraries were generated with TruSeq Stranded Total RNA Sample Prep Kit (Illumina) according to the manufacturer’s protocols. The libraries were then sequenced on an Illumina Hiseq2000 using a single-read protocol of 100 cycles with v3 chemistry at CAS-MPG Partner Institute for Computational Biology Omics Core, Shanghai, China.

**Cardiotoxin injury**

Cardiotoxin (CTX) was dissolved in sterile saline to a final concentration of 10 µM. Six to eight weeks of mice were anesthetized by isoflurane. Mouse legs were shaved and cleaned with alcohol. Tibialis anterior (TA) muscles were injected with 50 µL of CTX with a 26-gauge needle. The mice were sacrificed and the TA muscles were harvested at day 5 after CTX injection. Harvested muscles were frozen, cut at 10 µm and stained with hematoxylin and eosin.

**Hematoxylin-eosin staining**

Frozen transverse sections (10 µm) of TA containing region of CTX injury, deparaffinized in xylene, rehydrated through graded ethanol. Sections were subjected to hematoxylin staining for 7 minutes and eosin staining for 30 seconds.

**Supplemental Figure and Legends**

**
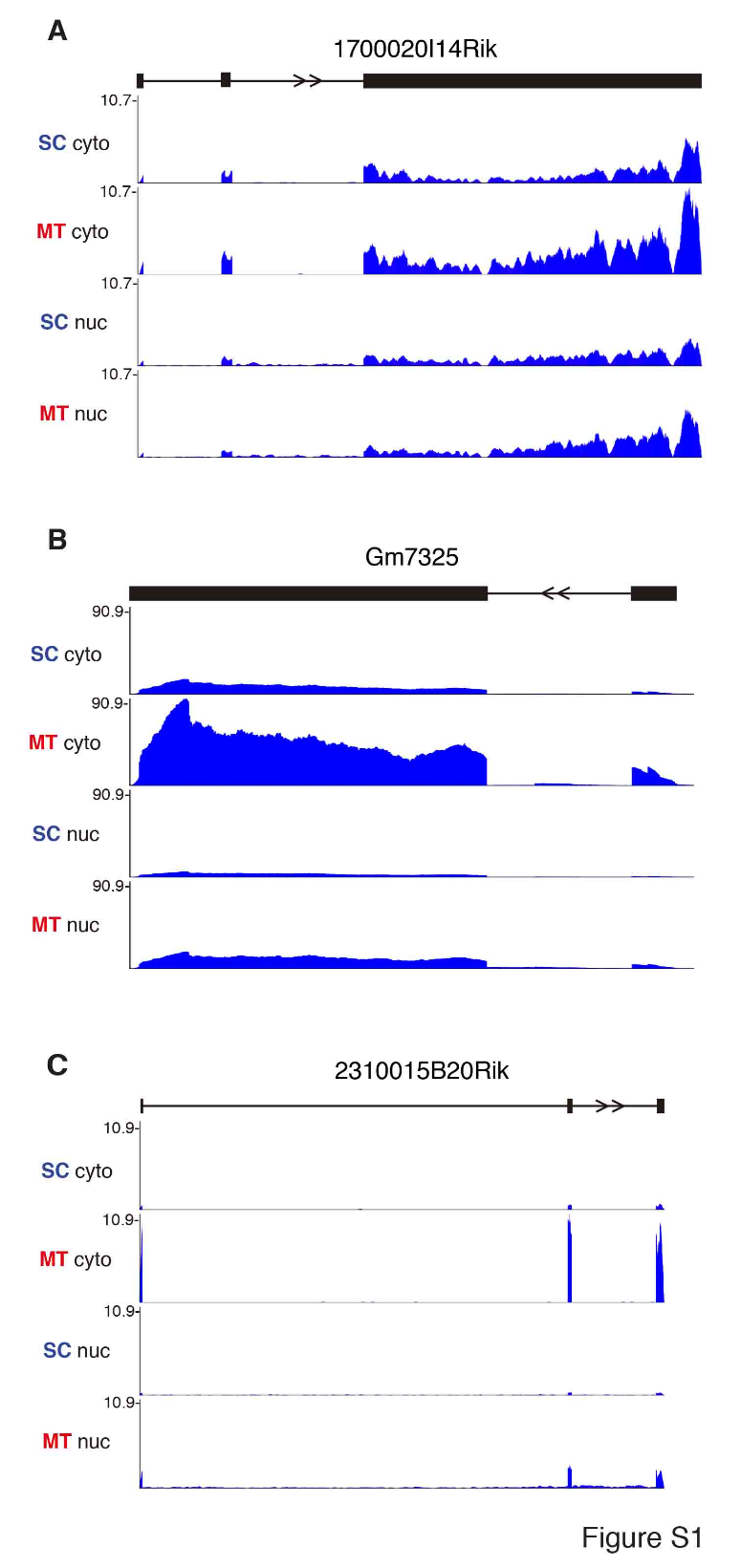
**

**Figure S1. RNA-seq signals for lncRNAs known to be enriched in myotubes.**

(**A-C**) Deep-sequencing signals of 1700020I14Rik (**A**), Gm7325 (**B**), and 2310015B20Rik (**C**). Numbers to the left show the RPM. SC, satellite cell; MT, myotube; cyto, cytoplasm; nuc, nucleus.


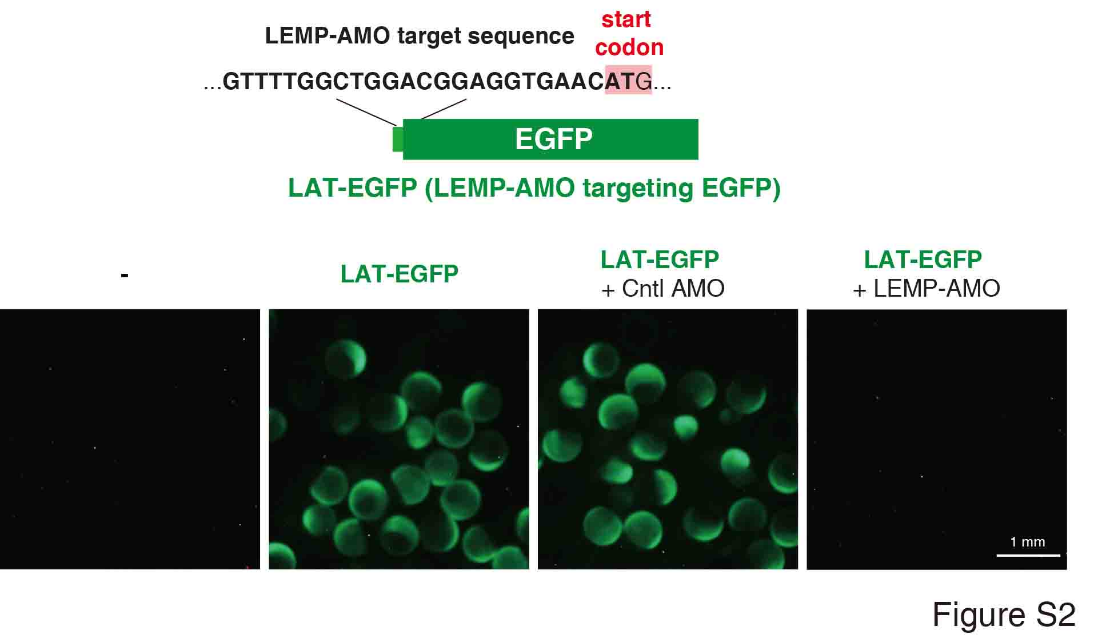


**Figure S2. Analysis of LEMP-specific morpholino efficiency.**

(Top): Schematic of LAT-EGFP (LEMP-AMO targeting EGFP) construct. (Bottom): GFP fluorescence signal of embryos at one-cell stage injected with LAT-EGFP mRNA, Cntl AMO/LAT-EGFP mRNA, LEMP AMO/LAT-EGFP mRNA respectively. Scale bar, 1 mm.

**Supplemental Figure Tables**

**Table S1** SiRNAs, AMO and sgRNAs used in this study

| **siRNAs used in this study** | |
| --- | --- |
| **Target gene** | **Targeting sequence (5’ to 3’)** |
| Cntl | AACAGUCGCGUUUGCUACUUU |
| MyolncR4-1# | TGTCCGTGCTAGTGGCTTT |
| MyolncR4-2# | GCACGGCTTGCAAGAACAT |
| **AMO used in this study** | |
| Cntl AMO | CCTCTTACCTCAGTTACAATTTATA |
| LEMP AMO | ATGTTCACCTCCGTCCAGCCAAAAC |
| **sgRNAs used in this study** | |
| LEMP-ORF-sgRNA | CACCGCTGAGAGGACGCTGCAGG |
| LEMP-Flag-KI-sgRNA | CACCGCCTGAGCACGCGCTGCAGCCCGA |
| LEMP-KO mouse 5’ sgRNA | TTTGTGCTGAGTGGTTTGCAATGG |
| LEMP-KO mouse 3’ sgRNA | GGGCTGGAGATGGATCACCTGGG |

**Table S2** Primers used in this study

| **RT-qPCR primers used in this study** | | |
| --- | --- | --- |
| GAPDH-F | Forward | AATAGGCGAACGCCTACTTC |
| GAPDH-R | Reverse | CCAGCTCTTTATTGAGATCAG |
| LEMP-F | Forward | CTGCGGAGGCGTTACC |
| LEMP-R | Reverse | CATCTTCGACAAGCTG |
| MHC-F | Forward | ATGCCACCTTCGCTACAACA |
| MHC-R | Reverse | GTTCAGCACTCGGTATCTCTGT |
